# Selected Papers from 2019 IEEE Eurasia Conference on Biomedical Engineering, Healthcare and Sustainability (IEEE ECBIOS 2019)

**DOI:** 10.3390/ijerph17082738

**Published:** 2020-04-16

**Authors:** Teen-Hang Meen, Yusuke Matsumoto, Kuei-Shu Hsu

**Affiliations:** 1 Department of Electronic Engineering, National Formosa University, Yunlin 632, Taiwan; thmeen@nfu.edu.tw; 2 Tokyo University of Pharmacy and Life Sciences, 1432-1 Horinouchi, Hachioji-city 192-0392, Tokyo, Japan; y-matsu@toyaku.ac.jp; 3 Department of Recreation and Health Care Management, Chia Nan University of Pharmacy & Science, Tainan City 71710, Taiwan

## Abstract

Recently, healthcare has undergone a sector-wide transformation thanks to advances in computing, networking technologies, big data, and artificial intelligence. Healthcare is not only changing from being reactive and hospital-centered to preventive and personalized, but it is also changing from being disease focused to well-being centered. Healthcare systems, as well as fundamental medicine research, are becoming smarter and enabled in biomedical engineering. This special issue on “Selected Papers from 2019 IEEE Eurasia Conference on Biomedical Engineering, Healthcare, and Sustainability (IEEE ECBIOS 2019)” selected nine excellent papers from 160 papers presented at IEEE ECBIOS 2019 on the topics of environmental health sciences and public health. Our aim is to encourage scientists to publish their experimental and theoretical research to promote scientific predictions and impact assessments of global change and development.

## 1. Introduction

The 2019 IEEE Eurasia Conference on Biomedical Engineering, Healthcare, and Sustainability (IEEE ECBIOS 2019) was held in Okinawa, Japan on 31 May–3 June 2019, and provided a unified communication platform for researchers in the fields of biomedical engineering, healthcare, and sustainability. Recently, healthcare has undergone a sector-wide transformation thanks to advances in computing, networking technologies, big data, and artificial intelligence. Healthcare is not only changing from being reactive and hospital-centered to preventive and personalized, but it is also changing from being disease focused to well-being centered. Healthcare systems, as well as fundamental medicine research, are becoming smarter and enabled in biomedical engineering. Furthermore, with cutting edge sensors and computer technologies, healthcare delivery could also yield better efficiency, higher quality, and lower cost. This special issue on “Selected Papers from 2019 IEEE Eurasia Conference on Biomedical Engineering, Healthcare, and Sustainability (IEEE ECBIOS 2019)” selected 9 excellent papers from 160 papers presented in IEEE ECBIOS 2019 on the topics of environmental health sciences and public health. It links several disciplines, including the environmental, economic, and social sustainability of human beings, which provide an advanced forum for studies related to environmental health sciences and public health. Our aim is to encourage scientists to publish their experimental and theoretical research to promote scientific predictions and impact assessments of global change and development. 

## 2. The Topics of Environmental Health Sciences and Public Health

This special issue selected nine excellent papers relative the topics of environmental health sciences and public health from 160 papers presented in IEEE ECBIOS 2019. The published papers are introduced as follows:

Tseng et al. reported “An Indoor Gardening Planting Table Game Design to Improve the Cognitive Performance of the Elderly with Mild and Moderate Dementia” [1]. The purpose of this study is to explore the potential needs and deficiencies of horticultural therapy activities in existing institutions, and to further develop indoor horticultural table games that can improve the overall cognitive function of the elderly with mild and moderate dementia. This study adopts the service experience insight method for its service design, with user experience as the core. By using the familiar horticultural activities of residents in Yunlin County, the elderly with mild and moderate dementia in Yunlin County were the core users.

Tu et al. reported “Investigating the Relationship between the Third Places and the Level of Happiness for Seniors in Taiwan” [2]. This study uses the questionnaire survey, and the data of this study were collected from October to November 2018 in Taichung City Central District. A questionnaire survey was conducted in several administrative agencies and participants were selected by random sampling among the over-55-year-old citizens who were already retired. An estimate of 90% confidence limits with 5% marginal error gave us a sample size of 257. This study finally received 200 efficient samples. The women’s top five choices of third places are the traditional market, supermarket, restaurant, daily necessities shop, and coffee shop. The men’s top five choices of third places are the traditional market, supermarket, daily necessities shop, restaurant, and a friend’s house. For seniors familiar with the concept of third places, the more often they go to third places, the higher happiness they achieve. This result investigates the importance of having awareness of third places for seniors. Therefore, we should encourage them to go to third places and engage in social activities frequently to achieve successful aging.

Lee et al. reported “The Process of Constructing a Health Tourism Destination Index” [3]. The purpose of the study is to identify a set of key indicators with weightings for health tourism destinations by using an advanced analytic hierarchy process (AHP) method, derived from the official, academic, and professional opinions of the experts. The AHP method allocated weightings to the evaluation criteria selected by the fifteen experts. After expert evaluations were conducted, the three dimensions and eleven sub-dimensions of the initial health tourism destination were obtained as follows: (1) special demands and indications—medical care, health promotion, and tourism and leisure; (2) natural environment—climate, air, water, and light; (3) leisure activities and general demands—sports, therapeutic activities, interactions with animals and plants, and diet. The results revealed that the dimensions of special demands and indications were given the most attention and that the sub-dimensions of sports promotion were the highest ranked by expert groups. The official and academic opinions suggested that health tourism destinations should focus on special demands and indications, while professionals tended to consider the natural environment as a primary concern. In particular, they considered that good air quality can help people release pressure, relax, activate lymphocytes, improve immune function, and enhance disease immunity. The health tourism destination index can contribute to the overall strategic planning process by identifying improvements in activities and enhancing competitiveness in health tourism management by using benchmarking to further improve tourists’ experience and satisfaction.

Chang et al. reported “Drama Therapy Counseling as Mental Health Care of College Students” [4]. This study aims to apply drama therapy to a counseling group to address the mental health problems of college students in Taiwan due to the increasingly serious psychological problems that have happened in recent times. Based on the healing factors in drama therapy, we applied such therapy activities to four counseling groups composed of 12 high-risk students from Taiwan. The results revealed that drama therapy could deliver significantly positive effects for and improve the six mental health indicators of the participants. Males’ self-awareness and decision-making actions were more positively affected than females. The study helps to provide a path for establishing the mental health module of drama therapy in the education sector in Taiwan.

Hwang et al. reported “Effects of Epiphytes and Depth on Seagrass Spectral Profiles: Case Study of Gulf St. Vincent, South Australia” [5]. The objectives in this study are to determine the influence of (1) epiphytes, (2) water depth, and (3) seagrass genus on the detection of reflectance spectral signals. The results show that epiphytes significantly dampen bottom-type reflectance throughout most of the visible light spectrum, excluding 670–679 nm; the depth does influence reflectance, with the detection of deeper seagrasses being easier, and as the depth increases, only Heterozostera increase in the exact “red edge” wavelength at which there is a rapid change in the near-infrared (NIR) spectrum. These findings helped improve the detection of seagrass endmembers during remote sensing, thereby helping protect the natural resource of seagrasses.

Cheng et al. reported “Training and Evaluation of Human Cardiorespiratory Endurance Based on a Fuzzy Algorithm” [6]. In this paper, we proposed a fuzzy system based on the human heart rate to provide an effective cardiorespiratory endurance training program and the evaluation of cardiorespiratory endurance levels. Trainers can respond correctly with the help of a smart fitness app to obtain the desired training results and prevent undesirable events such as under-training or over-training. The fuzzy algorithm, which is built for the Android mobile phone operating system receives the resting heart rate (RHR) of the participants via Bluetooth before exercise to determine the suitable training speed mode of a treadmill for the individual. The computer-based fuzzy program takes RHR and heart rate recovery (HRR) after exercise as inputs to calculate the cardiorespiratory endurance level. The experimental results show that, after eight weeks of exercise training, the RHR decreased by an average of 11%, the HRR increased by 51.5%, and the cardiorespiratory endurance evaluation level was also improved. The proposed system can be combined with other methods for fitness instructors to design a training program that is more suitable for individuals.

Wang et al. reported “Effect of Pharmacist Intervention on a Population in Taiwan with High Healthcare Utilization and Excessive Polypharmacy” [7]. This study investigated the changes in the number of medications, drug interactions and interaction severity in high frequency outpatients with polypharmacy at hospitals and clinics in Taiwan after home pharmaceutical care, to understand the effectiveness of interventions by pharmacists. Cases with excessive polypharmacy (10+ drugs) were selected from the Pharmaceutical Care Practice System database of the Taiwan Pharmacist Association in 2017. After the home care intervention, the number of drug types used decreased 1.89-fold (*p* < 0.001), and the number of medications fell 61.6%. The incidence of drug interaction was 93.82%. In an average case, the incidence of drug interaction after the pharmacist intervention decreased 0.6-fold (*p* < 0.001). The drug most commonly causing interactions was aspirin, followed by diclofenac; also common were three used in diabetes, two psycholeptics and two beta blockers. Among 22 cases of severe drug interaction, seven resulted in increased risk of extrapyramidal symptoms and neuroleptic malignant syndrome. By analyzing the relationship between the side effects of individual drugs and the pharmacokinetic T_max_, a sequential thermal zone model of adverse drug reactions can be established, the value of which could prompt physicians and pharmacists to intervene in order to prevent adverse events. It is concluded that home pharmaceutical care by pharmacists can significantly reduce the number of medications and interactions in patients with excessive polypharmacy and high healthcare utilization.

Wang et al. reported “Association between Potentially Inappropriate Medication Use and Chronic Diseases in the Elderly” [8]. The objective of this study was to investigate the continuous use of Potentially Inappropriate Medication (PIM) in a community-dwelling elderly population. A cross-sectional population-based study was conducted using community pharmacy-filed dispensing records from the Hcare system. Twenty-three community pharmacies were sampled from 2013 to 2015 to obtain records of patients above 65 years-old with continuous prescriptions. PIM were identified according to the 2015 Beers Criteria. The prevalence of patients using PIM was highest in patients with co-morbid mental disorders (40.05%), followed by neurological system disorders (28.91%). Patients who were prescribed a PIM were more than three times as likely to have a mental disorder as those (odds ratio 3.16, 95% confidence interval: 3.06–3.28) with non-chronic diseases. The most prescribed PIM agents were central nervous system drugs (53.16%), and benzodiazepines (35.15%). Patients with mental disorders had the highest rate of long-term persistent PIM exposure, with benzodiazepines being the most frequently dispensed. Drug safety concerns should be closely monitored in elderly patients with the abovementioned conditions.

Tu et al. reported “A Survey on Satisfaction of Type 2 Diabetes Patients with Different Demographic Variables to Medical Services” [9]. This study used the SPSS statistical software (IBM, Armonk, New York, NY, USA) for analysis, and the results show that: (1) Patients of different genders had different degrees of satisfaction with medical services. (2) The difference in age, monthly disposable income, occupational category, and education level had no significant effect on service satisfaction. (3) The research subjects were all on the high side regarding their satisfaction with the service provided by medical facilities. This study is a pilot study, and it is hoped it will be used as a guideline for improving patient care quality in the future, thus, reducing the occurrence of diabetic complications through better medical care. The long-term goal is to continuously improve care and medical service quality, thus reducing the waste of medical resources.
